# The Health Care Utilization and Medical Costs in Long-Term Follow-Up of Children Diagnosed With Leukemia, Solid Tumor, or Brain Tumor: Population-Based Study Using the National Health Insurance Claims Data

**DOI:** 10.2196/42350

**Published:** 2023-03-02

**Authors:** James S Miser, Ben-Chang Shia, Yi-Wei Kao, Yen-Lin Liu, Shih-Yen Chen, Wan-Ling Ho

**Affiliations:** 1 Cancer Center Taipei Medical University Hospital Taipei Taiwan; 2 Taipei Cancer Center Taipei Medical University Taipei Taiwan; 3 Department of Pediatrics City of Hope National Medical Center Duarte, CA United States; 4 Graduate Institute of Business Administration College of Management Fu Jen Catholic University New Taipei City Taiwan; 5 Artificial Intelligence Development Center Fu Jen Catholic University New Taipei City Taiwan; 6 Department of Applied Statistics and Information Science Ming Chuan University Taoyuan City Taiwan; 7 Department of Pediatrics School of Medicine, College of Medicine Taipei Medical University Taipei Taiwan; 8 Department of Pediatrics Taipei Medical University Hospital Taipei Taiwan; 9 Taipei Medical University Research Center of Cancer Translational Medicine Taipei Medical University Taipei Taiwan; 10 Division of Pediatric Gastroenterology, Department of Pediatrics Shuang Ho Hospital Ministry of Health and Welfare New Taipei Taiwan; 11 Department of Pediatrics Shin Kong Wu Ho-Su Memorial Hospital Taipei Taiwan

**Keywords:** brain tumor, cancer survivor, children, cost of care, health care, health resource, leukemia, long-term follow-up, population-based study, solid tumor

## Abstract

**Background:**

Childhood cancer survivors are at a high risk of medical consequences of their disease and treatment. There is growing information about the long-term health issues of childhood cancer survivors; however, there are very few studies describing the health care utilization and costs for this unique population. Understanding their utilization of health care services and costs will provide the basis for developing strategies to better serve these individuals and potentially reduce the cost.

**Objective:**

This study aims to determine the utilization of health services and costs for long-term survivors of childhood cancer in Taiwan.

**Methods:**

This is a nationwide, population-based, retrospective case-control study. We analyzed the claims data of the National Health Insurance that covers 99% of the Taiwanese population of 25.68 million. A total of 33,105 children had survived for at least 5 years after the first appearance of a diagnostic code of cancer or a benign brain tumor before the age of 18 years from 2000 to 2010 with follow-up to 2015. An age- and gender-matched control group of 64,754 individuals with no cancer was randomly selected for comparison. Utilization was compared between the cancer and no cancer groups by χ2 test. The annual medical expense was compared by the Mann-Whitney *U* test and Kruskal-Wallis rank-sum test.

**Results:**

At a median follow-up of 7 years, childhood cancer survivors utilized a significantly higher proportion of medical center, regional hospital, inpatient, and emergency services in contrast to no cancer individuals: 57.92% (19,174/33,105) versus 44.51% (28,825/64,754), 90.66% (30,014/33,105) versus 85.70% (55,493/64,754), 27.19% (9000/33,105) versus 20.31% (13,152/64,754), and 65.26% (21,604/33,105) versus 59.36% (38,441/64,754), respectively (all *P*<.001). The annual total expense (median, interquartile range) of childhood cancer survivors was significantly higher than that of the comparison group (US $285.56, US $161.78-US $535.80 per year vs US $203.90, US $118.98-US $347.55 per year; *P*<.001). Survivors with female gender, diagnosis before the age of 3 years, and diagnosis of brain cancer or a benign brain tumor had significantly higher annual outpatient expenses (all *P*<.001). Moreover, the analysis of outpatient medication costs showed that hormonal and neurological medications comprised the 2 largest costs in brain cancer and benign brain tumor survivors.

**Conclusions:**

Survivors of childhood cancer and a benign brain tumor had higher utilization of advanced health resources and higher costs of care. The design of the initial treatment plan minimizing long-term consequences, early intervention strategies, and survivorship programs have the potential to mitigate costs of late effects due to childhood cancer and its treatment.

## Introduction

The survival of children diagnosed with cancer has dramatically improved over the last 4 decades [1,2]. This improvement is primarily the result of advancements in the conventional treatment of the cancers with chemotherapy, surgery, and radiotherapy. Unfortunately, these therapeutic modalities have significant short- and long-term morbidities [3-5]. More recently, advances in the understanding of the biology of the cancers in children promise to allow more targeted therapy and tailored treatment regimens that will reduce the duration and toxicity of the therapy [6-8]. Unfortunately, these targeted therapies and tailored treatments are only beginning to be incorporated into the initial therapy of children with a malignant or brain tumor. Despite the 80% survival rate currently seen among children with cancer, a recurrence of the primary cancer remains the main important concern for most children with malignancy [1,2,9,10]. Some malignancies with good risk features and low stage at diagnosis have a very low recurrence rate and can be expected to be cured. Reduction in short- and long-term morbidities is a major strategy for these malignancies. Other malignancies have a higher recurrence rate and lower survival, and thus, require further improvement in therapy.

Disease- and therapy-related complications that result in long-term disabilities have increased over recent years as the cure rate has increased. An example of this is the physical disabilities associated with limb-sparing procedures for bone tumors as improvements in surgical techniques and equipment have been made. Further, as an increasing number of children are cured of brain tumors, many have long-term health issues including seizure disorders, other neurologic effects of the primary tumor [11-13], and endocrinologic abnormalities [14,15]. These complications are primarily cognitive and neurologic effects of chemotherapy and radiation therapy [11-13,16-18]; endocrinologic complications resulting from surgery, radiation therapy, alkylating agents, certain tyrosine kinase inhibitors, and immune system modulators [14,15,19-24]; organ system toxicity [22-32]; and psychologic and emotional effects [33-37].

Estimates [38,39] suggest that there are currently 16.9 million cancer survivors in the United States. The average out-of-pocket spending each year for cancer survivors aged 18-64 years was recently reported to be approximately US $1000, compared with US $622 for people without a history of cancer [38]. Although there is now a growing body of knowledge about the long-term effects on children who survived cancer, only little information is available on the long-term costs of health care for these children [40-45]. This is especially true for the long-term costs of health care not directly related to the primary cancer. This report evaluates the utilization of health care resources and their costs for long-term survivors of childhood cancer in Taiwan.

## Methods

### Research Database

We analyzed the national databases of the Health and Welfare Data Science Center (HWDC) in Taiwan including the death registry of the Taiwanese population and claims data of the National Health Insurance (NHI) from the Bureau of National Health Insurance. In Taiwan, the NHI is a single-payer compulsory social insurance plan enrolling more than 99% of its residents [46,47], a population of 25.68 million covered individuals at the end of 2005. After deidentification by the HWDC, the database is open to researchers in Taiwan for research applications.

### Study Design

This is a nationwide, population-based, case-control study of individuals who had a diagnostic code of cancer (ICD-9-CM [International Classification of Diseases, Ninth Revision, Clinical Modification] code 140-208) or a benign brain tumor (ICD-9-CM code 225) and who had survived for more than 5 years. The study included all survivors of childhood cancer who appeared in the national database between 2000 and 2010 and aged less than 18 years at the time of first appearance in the database. Survivors who were diagnosed between 1983 and 1999 were included in the data set as long as they were younger than 18 years when they first appeared in the database during years 2000-2010. The study population included cases whose birthday was as early as 1983 because they could have first appeared in the database beginning in the year 2000 at the age before 18. We defined the index date of follow-up as the date 5 years after the initial coding of cancer or a benign brain tumor in the claims data. To evaluate the costs of health care in long-term survivors of childhood cancer, we compared the utilization and costs of health care between children with cancer or a benign brain tumor who survived their disease more than five years and children without a history of cancer or brain tumor. Patients who died within 5 years of first diagnosis or first coding of cancer or a benign brain tumor in the database, who had no follow-up records beyond 1 year after the index date, or who had used antineoplastic and immunomodulating agents (Anatomical Therapeutic Chemical [ATC] class L) during the follow-up period were excluded from analysis.

### Case Group

The case group of childhood cancer survivors (cancer group) consisted of 33,105 patients who were diagnosed with cancer at an age less than 18 years. The age at entry to the study was defined by the year of first coding of cancer or a benign brain tumor in the database minus the birth year. All the survivors in the case group had at least 6 years of tracking records (5 years after initial appearance in the database plus 1 year of follow-up). The survivors were further classified as having/had hematologic cancer (ICD-9-CM codes 200-208), brain cancer (including all central nervous system [CNS] cancers; ICD-9-CM codes 191-192), benign brain tumor (including low-grade CNS tumors; ICD-9-CM code 225), and non-CNS solid tumor (ICD-9-CM codes 140-190 and 193-199).

The index date for the initiation of tracking health care utilization and costs was 5 years after the first date of cancer coding for each case and their matched comparison individuals. The date of the latest medical claims in the database was the endpoint of tracking health care utilization and costs.

### Comparison Group

An age- and gender-matched comparison group was included for comparison. To identify a matched comparison group, we randomly selected children without cancer or a benign brain tumor from the claims database; 2 comparison individuals were enrolled for each survivor of cancer or a benign brain tumor. A total of 64,754 comparison group individuals were selected by matching the propensity score of age and gender for each case [48,49]. As each survivor in the cancer groups was matched by 2 individuals with no cancer, the index date for each of the 2 individuals in the comparison group was set to be the same as their matched survivor in the cancer groups.

### Outcomes of Interest

The proportion of individuals utilizing health care services, annual outpatient visits, and the annual medical expense per person were calculated for each group. The annual medical expenses were the total medical cost between the index date and the last day of follow-up in this study divided by the years of follow-up. The utilization and cost in each domain of health care (ie, outpatient, inpatient, and emergency) and in each level of health care (ie, community clinic, regional hospital, and medical center) were calculated respectively and compared across groups.

### Statistical Analysis

The proportion of categorical variables such as sex, age, and health care utilization status was analyzed by the *χ*^2^ test. Continuous variables including age, years of follow-up, and annual medical expenditure were expressed as the median and IQR; the Mann-Whitney *U* test was used for 2-group comparisons and the Kruskal-Wallis rank-sum test for multiple-group analyses. We demonstrated the annual outpatient medical expenditures (by gender and stratified age groups) graphically by radar plots and box plots. The expenses were converted from Taiwan dollars to United States dollars at the exchange rate of 30:1. All data processing in this study was performed using SAS 9.3 (SAS Institute), and statistical analysis and graphing were performed using R 3.6.1 (R Foundation for Statistical Computing). Two-sided *P* values <.05 were considered statistically significant.

### Ethics Approval

This study was approved by the Joint Institutional Review Board of Taipei Medical University (TMU-JIRB N201911023), which agreed that informed consent can be waived when using these administrative data for analyses.

## Results

### Demographics

A cohort of 33,105 patients surviving 5 years after the first appearance of childhood cancer or a benign brain tumor in the database were identified (cancer group) and a comparison group of 64,754 individuals was selected from the deidentified national claims database of the HWDC in Taiwan from 2000 to 2015 (Table 1). The median follow-up starting from 5 years after the first appearance in the database (cancer group) or the index date of enrollment for the comparison group was 7 years. The cancer groups consisted predominantly of solid tumors (29,171/33,105, 88.12%); approximately two-thirds of the solid tumors were outside the CNS, whereas one-third originated in the CNS. The hematologic cancers comprised 11.88% (3934/33,105) of the cancer groups. The gender distribution of the cancer and no cancer groups was comparable with a slight male predominance (male:female=1.2:1) in both groups. The age distribution among the cancer and comparison groups was similar, with median ages between 11 and 14 years (Table 1).

**Table 1:** Demographics of the cancer and comparison groups.

Demographics		Childhood cancer survivors (n=33,105)	Cancer groups (ICD-9-CM^a^)						Comparison group	
			Hematologic cancer (200-208) (n=3934)	Brain cancer (191-192) (n=2241)	Benign brain tumor (225) (n=7825)	Non-CNS^b^ solid tumor (140-190; 193-199) (n=19,105)	*P* value^c^	No cancer individuals (n=64,754)		*P* value^d^
**Gender, n (%)**										
	Male	18,377 (55.51)	2211 (56.20)	1209 (53.95)	4091 (52.28)	10,866 (56.88)	<.001	35,803 (55.29)		.52
	Female	14,728 (44.49)	1723 (43.80)	1032 (46.05)	3734 (47.72)	8239 (43.12)	N/A^e^	28,951 (44.71)		N/A
Age at entry, median (IQR)		12.00 (7.00-16.00)	11.00 (5.00-15.00)	12.00 (8.00-16.00)	14.00 (8.00-17.00)	12.00 (6.00-16.00)	<.001	12.00 (6.00-16.00)		.002
Years of follow-up, median (IQR)		7.23 (3.58-10.01)	7.73 (4.73-10.53)	8.59 (5.25-10.90)	7.60 (4.73-10.00)	6.54 (3.07-9.76)	<.001	7.16 (3.53-9.98)		.04

^a^ICD-9-CM: International Classification of Diseases, Ninth Revision, Clinical Modification.

^b^CNS: central nervous system.

^c^Compared among the cancer groups and the no cancer group.

^d^Compared between all cancer survivors versus no cancer individuals.

^e^N/A: not applicable.

### Utilization of Medical Services

We examined the difference in medical service utilization between cancer survivors and no cancer individuals (Table 2). Cancer survivors had a higher utilization of medical center, regional hospital, inpatient, and emergency services in contrast to individuals without cancer: 57.92% (19,174/33,105) versus 44.51% (28,825/64,754), 90.66% (30,014/33,105) versus 85.70% (55,493/64,754), 27.19% (9000/33,105) versus 20.31% (13,152/64,754), and 65.26% (21,604/33,105) versus 59.36% (38,441/64,754), respectively (all *P*<.001), with more cancer survivors using medical center services (19,174/33,105, 57.92%, vs 28,825/64,754, 44.51%). We then examined the difference in service utilization among the cancer and no cancer groups. Brain cancer and benign brain tumor survivors demonstrated a different pattern of utilization, that is, more brain cancer and benign brain tumor survivors used inpatient and emergency services in contrast to hematologic and non-CNS solid tumor survivors. A higher proportion of benign and malignant brain tumor survivors also utilized secondary and tertiary care services, including hospital outpatient, inpatient, and emergency services. Of note, the utilization of emergency services was high among both the survivors and no cancer individuals (21,604/33,105, 65.26% and 38,411/64,754, 59.32%), reflecting societal patterns of seeking medical advice (Table 2).

**Table 2:** Utilization of medical services of the cancer and comparison groups.

			Study group^a^															
Category			Childhood cancer survivors (n=33,105), n (%)		Cancer groups (ICD-9-CM^b^)											Comparison group		
					Hematologic cancer (200-208) (n=3934), n (%)		Brain cancer (191-192) (n=2241), n (%)		Benign brain tumor (225) (n=7825), n (%)		Non-CNS^c^ solid tumor (140-190; 193-199) (n=19,105), n (%)		*P* value^d^		No cancer individuals (n=64,754), n (%)			*P* value^e^
**Outpatient**													.01					.001
	Yes	33,105 (100.0)		3934 (100.0)		2241 (100.0)		7825 (100.0)		19,105 (100.0)				64,721 (99.95)				
	No	0 (0.0)		0 (0.0)		0 (0.0)		0 (0.0)		0 (0.0)				33 (0.05)				
**Hospital outpatient**													<.001					<.001
	Yes	28,714 (86.74)		3476 (88.36)		2140 (95.49)		7193 (91.92)		15,905 (83.25)				51,846 (80.07)				
	No	4391 (13.26)		458 (11.64)		101 (4.51)		632 (8.08)		3200 (16.75)				12,908 (19.93)				
**Community clinic**													<.001					.002
	Yes	33,021 (99.75)		3926 (99.80)		2222 (99.15)		7804 (99.73)		19,069 (99.81)				64,509 (99.62)				
	No	84 (0.25)		8 (0.20)		19 (0.85)		21 (0.27)		36 (0.19)				245 (0.38)				
**Inpatient**													<.001					<.001
	Yes	9000 (27.19)		1060 (26.94)		848 (37.84)		2483 (31.73)		4609 (24.12)				13,152 (20.31)				
	No	24,105 (72.81)		2874 (73.06)		1393 (62.16)		5342 (68.27)		14,496 (75.88)				51,602 (79.69)				
**Emergency**													<.001					<.001
	Yes	21,604 (65.26)		2641 (67.13)		1619 (72.24)		5496 (70.24)		11,848 (62.02)				38,441 (59.36)				
	No	11,501 (34.74)		1293 (32.87)		622 (27.76)		2329 (29.76)		7257 (37.98)				26,313 (40.64)				
**Regional hospital**													<.001					<.001
	Yes	30,014 (90.66)		3616 (91.92)		2178 (97.19)		7417 (94.79)		16,803 (87.95)				55,493 (85.70)				
	No	3091 (9.34)		318 (8.08)		63 (2.81)		408 (5.21)		2302 (12.05)				9261 (14.30)				
**Medical center**													<.001					<.001
	Yes	19,174 (57.92)		2344 (59.58)		1630 (72.74)		5060 (64.66)		10,140 (53.08)				28,825 (44.51)				
	No	13,931 (42.08)		1590 (40.42)		611 (27.26)		2765 (35.34)		8965 (46.92)				35,929 (55.49)				

^a^The study group included only patients with utilization record.

^b^ICD-9-CM: International Classification of Diseases, Ninth Revision, Clinical Modification.

^c^CNS: central nervous system.

^d^Compared among the cancer and no cancer groups.

^e^Compared between all cancer survivors and no cancer individuals.

### Distribution of Annual Outpatient Visits

**Figure 1:**
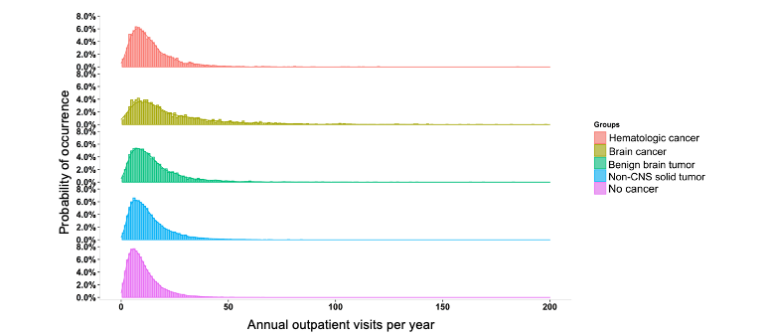
Distribution of annual outpatient visits in the cancer and comparison groups. The probability distribution of the annual outpatient visit frequencies in each group is summarized in a histogram. The area under the curve of each group is 100% of probability. Note the long tails in the brain cancer and benign brain tumor groups. CNS: central nervous system.

The outpatient visit frequency showed a markedly right-skewed distribution with a long tail in all the 4 cancer groups and the comparison group, suggesting that there were a number of individuals in each group with high utilization of outpatient services (Figure 1). The median frequency (IQR) of annual outpatient visits of all childhood cancer survivors was 11.38 (6.88-18.27) visits/year, which was significantly higher than 8.63 (5.20-13.71) visits/year of the comparison group (*P*<.001). The median frequency (IQR) of outpatient visits of the 4 cancer groups was different from that of the comparison group: 11.20 (6.95-17.46) for hematologic cancer, 16.54 (9.22-30.60) for brain cancer, 12.33 (7.29-19.69) for a benign brain tumor, and 10.72 (6.52-17.03) for a non-CNS solid tumor (*P*<.001). The shape of the distribution curve was different among the cancer groups; the long tails of the distribution curves tapered off more gradually in the brain cancer and benign brain tumor groups. The results suggest that the frequencies of annual outpatient visits were the highest in the brain cancer and benign brain tumor groups, and that there were some extreme users in these 2 groups (Figure 1).

### Medical Expense

The cost of medical services for childhood cancer survivors was higher than that for the comparison group (Table 3). The median total annual expense was 1.4-fold higher in childhood cancer survivors than in the comparison group (US $285.56, IQR US $161.78-US $535.80 per year vs US $203.90, IQR US $118.98-US $347.55 per year; *P*<.001); the median annual hospital outpatient cost and community clinic cost were also higher, 2.48- and 1.15-fold, respectively (*P*<.001). Moreover, childhood cancer survivors showed a different pattern of costs, with higher median annual expenses in medical centers (US $6.35 vs US $0 per year; *P*<.001) and regional hospitals (2.09-fold; *P*<.001) in contrast to no cancer individuals. Among the cancer groups, brain cancer and benign brain tumor survivors had a different pattern of costs, with higher median annual total and outpatient expenses, especially in emergency services (2.69- and 1.98-fold more over the comparison group, respectively; *P*<.001) and medical center services (US $68.37 vs US $0 and US $13.52 vs US $0 per year; *P*<.001) compared with hematologic and non-CNS solid tumor survivors (Table 3).

**Table 3:** Annual medical expense of the cancer and comparison groups.^a^

		Study group								
Category		Childhood cancer survivors (n=33,105)	Cancer groups (ICD-9-CM^b^)						Comparison group	
			Hematologic cancer (200-208) (n=3934)	Brain cancer (191-192) (n=2241)	Benign brain tumor (225) (n=7825)	Non-CNS^c^ solid tumor (140-190; 193-199) (n=19,105)	*P* value^d^	No cancer individuals (n=64,754)		*P* value^e^
Total expense		285.56 (161.78-535.80)	268.48 (159.50-475.08)	614.08 (267.05-1793.94)	336.89 (184.64-652.14)	257.78 (148.44-456.88)	<.001	203.90 (118.98-347.55)		<.001
**Outpatient**		239.81 (140.24-416.02)	230.96 (138.51-368.64)	473.60 (223.04-1380.48)	274.76 (159.54-503.51)	219.49 (129.69-362.93)	<.001	174.50 (103.38-280.90)		<.001
	Hospital outpatient	50.94 (10.46-165.40)	45.48 (10.93-132.53)	227.17 (42.62-1076.80)	75.48 (18.91-236.87)	39.37 (6.98-124.60)	<.001	20.56 (3.19-67.82)		<.001
	Community clinic	150.78 (89.09-237.73)	148.16 (92.35-232.70)	172.90 (93.45-284.81)	156.97 (91.28-248.53)	146.74 (87.16-230.49)	<.001	131.03 (76.53-206.54)		<.001
Inpatient		0.00 (0.00-36.49)	0.00 (0.00-30.92)	0.00 (0.00-142.36)	0.00 (0.00-72.10)	0.00 (0.00-0.00)	<.001	0.00 (0.00-0.00)		<.001
Emergency		8.45 (0.00-26.50)	7.87 (0.00-23.44)	14.29 (0.00-44.84)	10.54 (0.00-32.22)	7.25 (0.00-23.59)	<.001	5.31 (0.00-19.02)		<.001
Regional hospital		71.91 (19.90-235.61)	61.90 (18.78-183.95)	278.41 (64.85-946.18)	107.37 (30.41-314.84)	57.08 (15.07-178.88)	<.001	34.40 (8.50-109.28)		<.001
Medical center		6.35 (0.00-67.90)	6.93 (0.00-60.93)	68.37 (0.00-598.16)	13.52 (0.00-107.51)	2.82 (0.00-44.52)	<.001	0.00 (0.00-17.78)		<.001

^a^Data are presented as median expense in US $ per person per year (IQR).

^b^ICD-9-CM: International Classification of Diseases, Ninth Revision, Clinical Modification.

^c^CNS: central nervous system.

^d^Compared among the cancer groups and the no cancer group.

^e^Compared between all cancer survivors versus no cancer individuals.

### Annual Outpatient Expense

**Figure 2:**
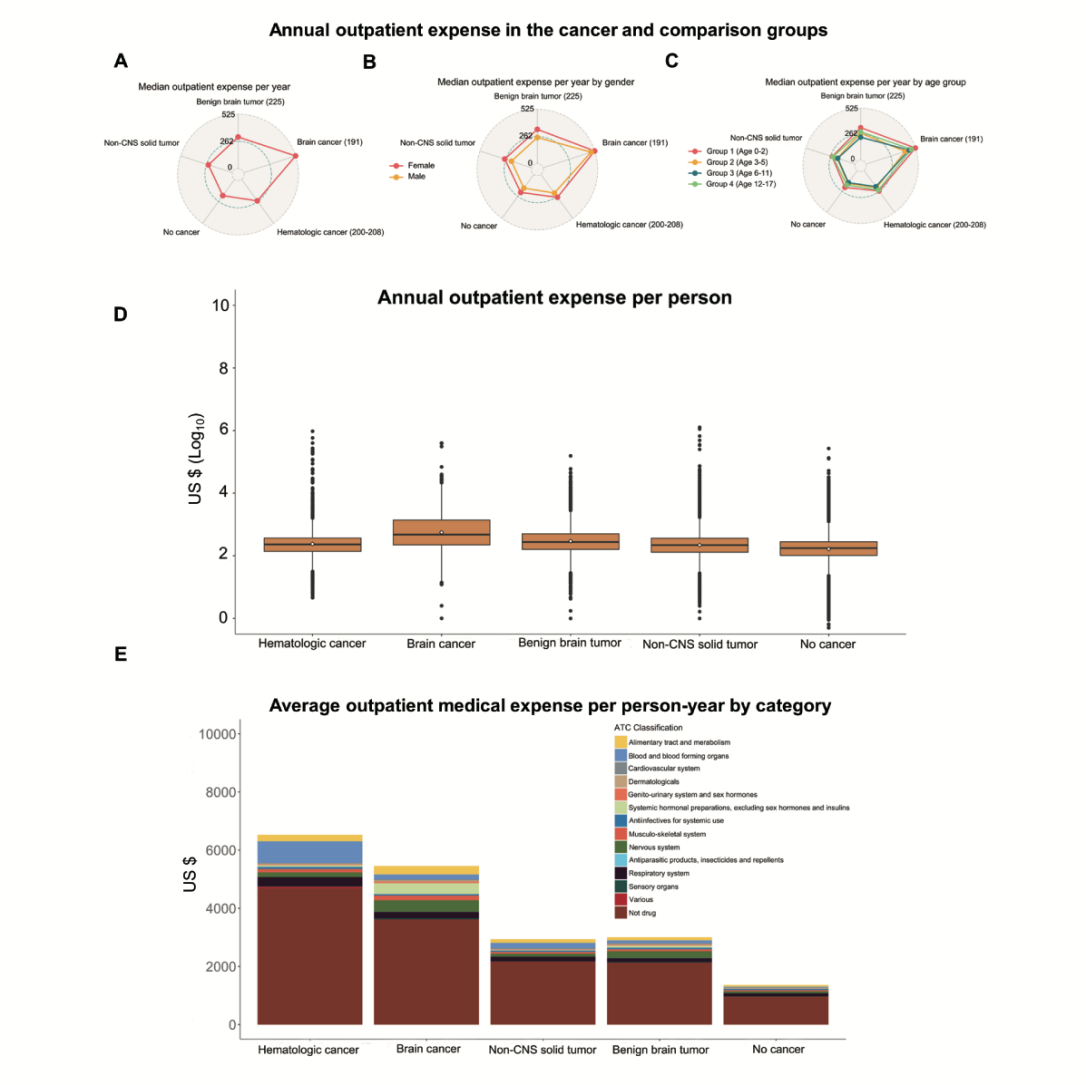
The median annual outpatient expense in each cancer and comparison group is shown (A) and compared by gender (B) and age at entry (C). The distribution of annual outpatient expense per person was compared across the cancer and comparison groups in a box plot (D) (circles, mean annual outpatient expense; boxes, the 25th, 50th, and 75th percentile of each group; solid vertical lines, 1.5 box length, ie, 1.5 interquartile range; dots, outliers). The average outpatient medical expense per person-year in each cancer and comparison group was compared (E). Color bars represent the Anatomical Therapeutic Chemical (ATC) Classification of medications and the lowest brown bars represent nonmedication costs of medical services. CNS: central nervous system.

**Figure 3:**
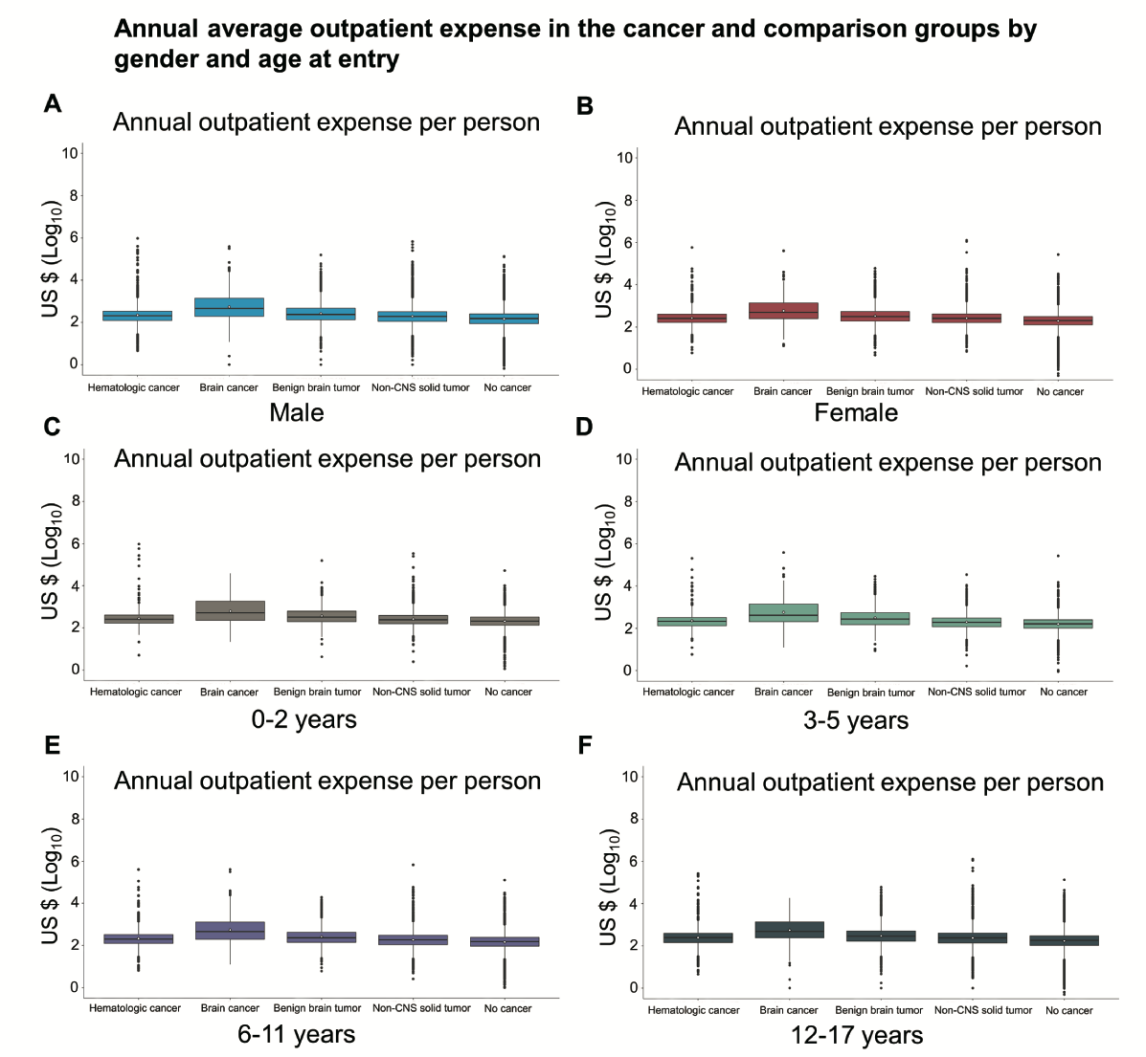
The annual outpatient expense in each cancer and comparison group was compared by male (A), female (B) and the age at entry of 0-2 years (C), 3-5 years (D), 6-11 years (E) and 12-17 years (F). Circles, mean annual outpatient expense; boxes, the 25th, 50th, and 75th percentile of each group; solid vertical lines, 1.5 box length, i.e., 1.5 interquartile range; dots, outliers.

The median outpatient expense per person of each cancer group was higher than that of the comparison group. Importantly, the median outpatient expense of the brain cancer group was approximately 2 times higher than that of the other cancer groups (Figure 2A). The pattern of annual outpatient expense of the cancer and comparison groups was the same for both genders (Figures 2B and 3A and 3B). To further examine the annual outpatient expense across different ages at entry, we stratified the age into 4 intervals, namely, 0-2, 3-5, 6-11, and 12-17 years. We found that the pattern of annual outpatient expense of the cancer and comparison groups was the same across all age groups (Figures 2C and 3C-3F). Interestingly, female childhood cancer survivors and comparison individuals had a higher annual outpatient expense than their male counterparts (Table 4). In addition, infants and children up to the age of 2 years had a higher annual outpatient expense than older individuals (Table 5). The annual outpatient expense of brain cancer survivors was similar across gender and ages; however, the brain cancer survivors had a higher annual outpatient expense compared with the other cancer groups and with the no cancer group across both genders and all ages at entry (Tables 4 and 5).

Brain cancer survivors had the highest mean and median annual outpatient expenses, in contrast to other cancer groups and the comparison group (*P*<.001). The IQR of outpatient expense per person per year of the brain cancer group was greater than that of the other cancer groups and the no cancer group (Figure 2D).

**Table 4:** Annual outpatient expense compared by gender.^a^

Gender	Study group											
	Cancer groups (ICD-9-CM^b^)									Comparison group		
	Hematologic cancer (200-208) (n=3934)	*P* value^c^	Brain cancer (191-192) (n=2241)	*P* value^c^	Benign brain tumor (225) (n=7825)	*P* value^c^	Non-CNS^d^ solid tumor (140-190; 193-199) (n=19,105)	*P* value^c^	No cancer individuals (n=64,754)		*P* value^c^	
Female	255.92 (163.12-401.30)	N/A^e^	492.35 (250.84-1364.01)	N/A	311.17 (189.30-538.72)	N/A	256.09 (160.60-410.86)	N/A	201.67 (126.46-309.05)		N/A	
Male	208.57 (123.75-342.92)	<.001	458.34 (193.74-1404.03)	.06	237.66 (134.68-469.39)	<.001	192.07 (111.78-322.92)	<.001	153.35 (88.48-252.09)		<.001	

^a^Data represent the median expense in US $ per person per year (IQR).

^b^ICD-9-CM: International Classification of Diseases, Ninth Revision, Clinical Modification.

^c^Compared with females in each group.

^d^CNS: central nervous system.

^e^N/A: not applicable.

**Table 5:** Annual outpatient expense compared by age at entry.^a^

Age at entry (years)	Study groups										
	Cancer groups (ICD-9-CM^b^)									Comparison group	
	Hematologic cancer (200-208) (n=3934)	*P* value^c^	Brain cancer (191-192) (n=2241)	*P* value^c^	Benign brain tumor (225) (n=7825)	*P* value^c^	Non-CNS^d^ solid tumor (140-190; 193-199) (n=19,105)	*P* value^c^	No cancer individuals (n=64,754)		*P* value^c^
0-2	257.74 (166.83-408.19)	Reference	525.27 (228.69-1847.69)	Reference	329.36 (197.63-621.16)	Reference	245.14 (157.84-393.75)	Reference	212.38 (136.58-329.15)		Reference
3-5	251.85 (133.05-323.90)	<.001	415.33 (204.98-1397.82)	.33	272.84 (151.28-550.89)	<.001	191.24 (118.96-305.10)	<.001	162.35 (102.46-251.89)		<.001
6-11	202.41 (126.32-330.68)	<.001	457.92 (197.78-1303.71)	.25	231.84 (140.18-419.25)	<.001	186.55 (108.48-306.03)	<.001	152.34 (92.1-239.84)		<.001
12-17	245.39 (142.83-386.15)	.01	481.73 (240.08-1364.01)	.53	286.93 (165.13-507.47)	<.001	238.28 (138.16-399.03)	<.001	182.10 (103.66-296.48)		<.001

^a^Data represent the median expense in US $ per person per year (IQR).

^b^ICD-9-CM: International Classification of Diseases, Ninth Revision, Clinical Modification.

^c^Compared with the age of 0-2 years in each group.

^d^CNS: central nervous system.

**Figure 4:**
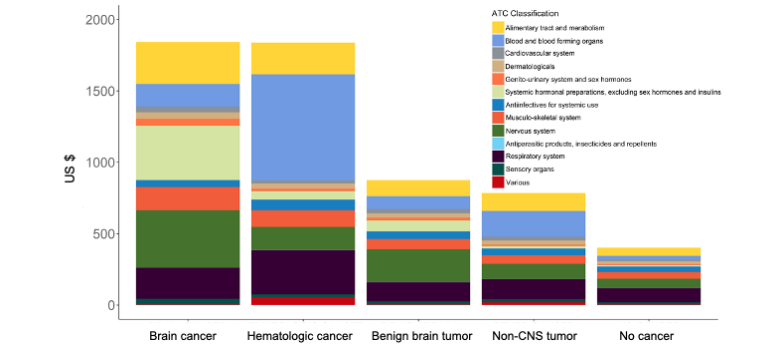
The average outpatient medical expense of medications per person-year in each cancer and comparison group was compared. Color bars represent the ATC Classification of medications. ATC: Anatomical Therapeutic Chemical.

To evaluate the composition of cost, we calculated the average outpatient medical expense per person-year of the cancer groups that was much higher than that of the no cancer group (Figure 2E). The hematologic and brain cancer groups had the highest average outpatient medical expense per person-year (4.79- and 4.01-fold of the no cancer group, respectively). The average outpatient medical expense per person-year of nonmedication service of hematologic and brain cancers was much higher (Figure 2E, brown bars). Moreover, the average outpatient medication expense per person-year was the highest in hematologic and brain cancers (Figure 4). Further, the cost was lower in medication than nonmedication services across all groups (Figure 2E). The proportion of medication cost was significantly higher in brain cancer survivors compared with no cancer individuals (33.79% vs 29.55%; *P*=.003), whereas the proportion of medication cost for survivors of hematological cancer (28.18% vs 29.55%; *P*=.31), benign brain tumor (29.16% vs 29.55%; *P*=.79), and non-CNS solid tumor (26.75% vs 29.55%; *P*=.06) was not significantly different than in the no cancer group (Figure 2E). In the brain cancer and benign brain tumor groups, medication costs for hormonal therapy, nervous system, alimentary tract, and metabolism were the major classifications of medication costs (Figures 2E and 4).

## Discussion

### Principal Findings

In children aged 0-14 years, the annual incidence of newly diagnosed cancers (excluding benign CNS tumors) is 178 cases/million in the United States and 125 cases/million in Taiwan [50-52]. With the advent of multidisciplinary care and multimodality treatments, the 5-year survival rate of childhood cancer has now exceeded 80% [1,2], especially with leukemias and lymphomas [53-55]. Because of this success, every year the number of childhood cancer survivors continues to rise. It is now estimated that in the next 10-20 years 1/1000 individuals in Taiwan aged 20-30 years will be a survivor of childhood cancer. In the United States, the number of individuals surviving their cancer and treatments is projected to reach more than 22.1 million by 2030 [38,39].

It has been reported that 62% of survivors of childhood cancer develop a long-term health problem and 27% have a serious or life-threatening condition [56]. As a result, only 1 in 3 survivors of childhood cancer remains free of long-term health problems related to the cancer and its treatment. Thus, the treatment outcomes of childhood cancer should be not only the survival rate but also the long-term health care issues that include medical service utilization and the medical costs of the services.

There is growing information about the long-term health issues of childhood cancer survivors; however, there are very few studies describing the utilization and costs of health care for this unique population. Although the average cost of childhood cancer treatment varies among countries, estimated to be US $32,157 in Korea [57-60], the long-term health care costs of survivors remain unknown. Our data demonstrate that the utilization and costs of medical service during long-term follow-up for childhood cancer survivors are greater than those for the comparison group without a previous cancer diagnosis. In addition, the patterns of health care services that are utilized by the cancer survivors and individuals without a history of cancer are very different, with cancer survivors utilizing more hospital care and advanced care.

Comparing childhood cancer survivors with individuals without a diagnosis of cancer, the former not only had higher utilization of health care but also had much higher medication and nonmedication costs. Although the cancer survivors had much higher medication cost, the main categories of medication costs of both groups were respiratory, gastrointestinal, and neurological. The nonmedication costs were the major component of costs in both groups.

When we evaluated the health care utilization and cost of each cancer group, it was clear that children with both malignant and benign brain tumors had unique medical needs in long-term follow-ups. Specifically, brain cancer survivors had the highest utilization and costs; similarly, benign brain tumor survivors also had high utilization and costs. This was also seen in a recent publication from the French Childhood Cancer Survivor Study and the French Network of Cancer Registries where the highest median annual health care expenditure was seen in survivors of a childhood CNS tumor [61]. Regarding outpatient medication costs for these subgroups, hormonal and neurological medications comprised the 2 largest proportions of costs in brain cancer and benign brain tumor survivors. Notwithstanding, all groups of survivors have an increased annual medical cost during long-term follow-up compared with the no cancer group.

In the analysis of costs, we noted that females had significantly higher costs than their male counterparts in both the cancer survivor and no cancer groups, similar to the findings from the French study [61]. The difference was especially large in survivors of a benign brain tumor. However, the cause of cost disparities between genders could not be determined by our data and thus needs further investigation.

Our data also suggest that cancer survivors who were diagnosed at younger ages, especially those with brain tumors, will require more services in their long-term care. These data are similar to those from the French study, where the annual health care expenditures were higher in children diagnosed at older ages or with a CNS tumor [61]. Likewise, in our study, the higher cost associated with brain cancer was seen in all age groups. To address the unique medical needs of these patients, a specialized, multidisciplinary team with individualized surveillance programs needs to be established to promote health and prevent future illness. Our data also support the attempt to mitigate the long-term effects of very young children with brain tumors by reducing, delaying, or eliminating radiation therapy [16-18].

Further study is needed to define the health issues that contribute to the increased health care cost and health care burden of childhood cancer survivors. The goals for the health care system are (1) to understand the extent of the health issues that survivors of childhood cancer face; (2) to understand the specific types of health issues that survivors of childhood cancer face; (3) to develop interventions to improve the health of the survivors of childhood cancer; (4) to decrease the health care costs of survivors of childhood cancer; and (5) to reduce the health care burden of survivors of childhood cancer to the health system. The goals for the survivors of childhood cancer are (1) to mitigate the extent of their health problems; (2) to mitigate the severity of their health problems; and (3) to improve their quality of life. Further, the design of the treatment plan for children with cancer needs to minimize long-term consequences. In addition, early intervention strategies to overcome potential disabilities caused by the cancer should be implemented at the time of diagnosis.

### Strengths and Limitations

There are several limitations of this study. First, this is a retrospective analysis of childhood cancer survivors in Taiwan. The proportion of hematologic cancer survivors is smaller than that of other survivorship cohorts [9,10], probably due to the relatively lower survival rate of those with acute leukemias in Taiwan during the early years of the study [62,63]. Second, we used claims data that were not directly linked to the nationwide cancer registry. The ICD-9 diagnostic codes, which were determined by physicians, could not be validated. We believe there were also a number of benign brain tumors representing tumors that are not typically considered in the benign brain tumor registries. We also believe there were a large number of benign solid tumors included in the “non-CNS solid tumor” group, which was intended to only include malignant diseases. In addition, the specific causes of costs for each cancer group could not be defined in this study and will require further analysis. Further, the history of treatment with stem cell transplantation or radiotherapy, which is known to be associated with late effects and increased medical costs, could not be analyzed in this study. The effects of these therapies should thus be evaluated in future studies. The strengths of this study are that it is a nationwide, population-based design; that the study cohort is relatively large, with the case group of childhood cancer survivors; and that there is a comparison group determined by matching the propensity score of age and gender from the claims data of the NHI that covers 99% of the Taiwanese population. Therefore, the results represent a real-world scenario, rather than assumptions and projections.

### Conclusions

The cost of health care of children in Taiwan surviving childhood cancer or a brain tumor was higher than that of an age- and gender-matched comparison group of children without a history of cancer. The utilization of health care resources among children in Taiwan surviving childhood cancer and its treatment was also higher than that among an age- and gender-matched comparison group without a history of cancer. The types of health care expenditures and issues for children surviving a malignant or a benign brain tumor were different from those of the normal population. Thus, these children require coordinated follow-up and comprehensive, specialized care to optimize outcomes and quality of life. To reduce these health care costs and utilization, a careful prospective analysis of the costs and patterns of utilization will be needed. Further, preventive strategies with effective interventions will be required.

## Abbreviations

ATC: Anatomical Therapeutic Chemical
CNS: central nervous system
HWDC: Health and Welfare Data Science Center
ICD-9-CM: International Classification of Diseases, Ninth Revision, Clinical Modification
NHI: National Health Insurance
